# Arabic version of the SF‐Qualiveen: cross-cultural adaptation, translation, and validation of urinary disorder‐specific instruments in patients with spinal cord injury

**DOI:** 10.1186/s13018-023-04411-2

**Published:** 2024-01-12

**Authors:** Younes A. Khadour, Bashar Ebrahem, Fater A. Khadour

**Affiliations:** 1 Department of Physical Therapy, Cairo University, Cairo, 11835 Egypt; 2 Department of Rehabilitation, Faculty of Medicine, Al Baath University, Homs, Syria; 3 Department of Physical Therapy, Health Science Faculty, Al-Baath University, Homs, Syria; 4 Department of Rehabilitation, Tongji Hospital, Tongji Medical College, Huazhong University of Science and Technology, 1095#, Jie-Fang Avenue, Qiaokou District, Wuhan, 430030 Hubei China

**Keywords:** The SF‐Qualiveen questionnaire, Quality of life, Neurogenic lower urinary tract dysfunction, Reliability, Validity

## Abstract

**Background:**

The Short-Form Qualiveen (SF-Qualiveen) questionnaire assesses the effect of bladder and urinary symptoms on patients' quality of life (QoL) with urological impairment caused by neurological diseases. There is no validated SF-Qualiveen questionnaire in Arabic, so this study aims to provide a translated and validated version of the SF-Qualiveen questionnaire among Arabic patients experiencing spinal cord injury (SCI).

**Methods:**

Psychometric features such as content and construct validity, test–retest reliability, and internal consistency were analyzed. Construct validity was evaluated by contrasting the SF-Qualiveen with the Neurogenic Bladder Symptom Score Short-Form (NBSS-SF) questionnaire. Internal consistency was measured using Cronbach's alpha, whereas the intraclass correlation coefficient (ICC) was employed to assess the test–retest reliability. Factorial validity was established by principal component analysis (PCA).

**Results:**

The internal consistency of the total SF-Qualiveen and the domains “Bother with limitations,” “Fear,” “Feeling,” and “Frequency of limitations” showed good internal consistency (Cronbach's alpha of > 0.7). ICC was 0.90 for the total score, 0.83 for the bother with limitations, 0.80 for fears, 0.84 for feeling, and 0.81 for frequency of limitations. The correlation analysis revealed a positive association between the total scores on the NBSS-SF and the domains of the SF-Qualiveen, comprising bother with limitations (*r* = 0.53, *p* = 0.02), fears (*r* = 0.44, *p* = 0.03), feelings (*r* = 0.49, *p* = 0.04), and frequency of limitations (*r* = 0.46, *p* = 0.02). The best-fit four-factor model for confirming overall item communalities ranged from 0.552 to 0.814, which indicates moderate to high communalities, and confirms the homogeneity of the SF-Qualiveen using PCA.

**Conclusions:**

The findings of this validation study revealed that the SF-Qualiveen is a reliable and valid instrument appropriate for Arabic-speaking patients with SCI in both research and clinical practices.

## Introduction

Neurogenic lower urinary tract dysfunction (NLUTD) induced by a disease of the nervous system is one of the more difficult disorders for health-care professionals to address [1]. Lower urinary symptoms are a frequent occurrence among spinal cord injury (SCI) patients, where more than 80% of patients with SCI experience bladder dysfunction or lower urinary tract symptoms [2]. Lower urinary tract dysfunction symptoms typically worsen and become more challenging to treat as disease duration and physical impairment increase [3]. This causes a considerable decline in SCI patients' quality of life (QoL).

As variations were observed between the perceptions of patients and doctors regarding the effect of neurological conditions like SCI, direct assessments of the patient's suffering and experiences are required to learn about their impression of the disease's effect on the quality of life [2]. The European Association of Urology (EAU) recommendations on neuro-urology emphasize the importance of quality of life in treating patients' neuro-urological. The urological condition of NLUTD patients with neurogenic lower urinary tract impairment ought to be carefully assessed. The assessment must involve a physical examination, urine testing, and ultrasonic and urodynamic investigations.

Urologists frequently utilize bladder diaries and several self-administered questionnaires to get a more precise and comprehensive record of symptoms of lower urinary tract dysfunction patients. The European Association of Urology (EAU) advises using a validated instrument to assess the quality of life of NDLUT patients [4]. More than thirty validated questionnaires are designed to assess the symptoms of urinary tract dysfunction and their effect on a patient's QoL. Only a few of these questionnaires were specially designed for patients with NLUTD [5]; out of them, the Qualiveen [6] and its short form (SF‐Qualiveen) [7] are the two commonly utilized questionnaires for assessing urinary-specific patients' QoL with SCI.

The Qualiveen was first validated and translated into English [8], then German [9], Italian [10], Portuguese [11], Spanish [12], and Turkish [13]. The Qualiveen was designed employing expert opinion, interviews with patients, and a literature review. It consists of 30 items in four domains: bother with limitations, fears, feelings, and frequency of limitations. Later, in 2008, Bonniaud et al. designed a short version of Qualiveen that includes eight questions assessing the four same domains as the Qualiveen version [14]. The SF-Qualiveen questionnaire is currently available in English [14], German [15], and Polish [16] for multiple sclerosis (MS) patients, but it has not yet been adapted for SCI patients. Although there are similarities in the neuro-urological dysfunction experienced by both MS and SCI patients, there are also differences in how these conditions are clinically present and how they impact the quality of life. For instance, SCI typically has an acute onset, whereas MS is a progressive disease.

Additionally, SCI often leads to a complete loss of sensation in the lower body, while multiple sclerosis (MS) may result in altered sensation but not a complete loss. Therefore, it is crucial to assess the validity and reliability of the SF-Qualiveen questionnaire among SCI patients in order to determine its effectiveness as a measurement tool for managing the quality of life of Arabic SCI patients. Because concerted efforts are needed to boost NLUTD patient outcomes and their quality of life, this study aimed to validate and culturally adapt an Arabic version of the SF-Qualiveen for use in SCI patients in clinical and research applications.

## Methods

This study is divided into two stages. The first one involves translating and cross-culturally adapting the SF-Qualiveen into Arabic. The second one involves assessing the Arabic SF-Qualiveen's validity and reliability.

### Stage 1: translation and cross-cultural adaptation

The SF-Qualiveen questionnaire has been translated using Beaton et al.'s self-reported measurement recommendations [17]. The forward translation of the English version of SF-Qualiveen into Arabic was completed independently by two bilingual translators, both of whose first language is Arabic. Then, a first consensus meeting was followed between the two translators and the principal researcher (FK). The consensus form was back-translated from Arabic into English by a similarly certified English translator in Arabic who was unaware of the study's goal or the original versions of the translated questionnaire. The Arabic and English translators and the principal researcher concurred during the second consensus meeting that the original and the Arabic versions were both understandable and equivalent. The final Arabic version was examined by a bilingual panel comprised of two neurorehabilitation specialists (AG and YR), a specialist in the neurologic bladder (AM), and one urogynecologist (SF). The Arabic version of the questionnaire was revised by the four staff members, who also approved it. In addition to evaluating each instrument item's semantic, idiomatic, and conceptual equivalence, the questionnaire items were reviewed to ascertain their clinical relevance and acceptability for usage in the specific patient group [17].

### Stage 2: validation

#### Reliability

The psychometric properties of the SF-Qualiveen questionnaire were evaluated under the principles of cross-cultural adaptation [17]. Reliability refers to the level of measurement accuracy [17]. This study evaluated both test–retest and internal consistency reliability [18]. Internal consistency is the degree to which items from the same domain correlate. Cronbach's alpha measures internal consistency, and a score greater than 0.70 can be considered good [18]. The test–retest of the SF-Qualiveen was assessed utilizing the SF-Qualiveen scores from visits 1 and 2. The intraclass correlation coefficient (ICC) was employed to assess the results. An association of more than 0.7 is regarded as strong [19].

### Validity

#### Construct and content validity

Validity indicates the precision of a measure (if the findings actually reflect what they are trying to measure) [18]. In this study, we investigated the content and construct validity. Construct validity refers to an instrument's capability to evaluate the underlying concept it is looking for [19]. This study determined construct validity by evaluating the correlation between the SF-Qualiveen and the Neurogenic Bladder Symptom Score Short-Form (NBSS-SF) questionnaire using Spearman's correlation coefficient. Content validity indicates how well an assessment tool (like a test) covers all relevant items of the construct it aims to measure. It can be assessed by specialists who decide whether the proposed items on the instrument are relevant to the phenomenon to be assessed [19]. Content validity was evaluated through patient interviews; thirty Arabic-speaking SCI were asked to evaluate the content validity in face-to-face interviews during September and October 2022. These patients were requested to fill out a questionnaire first. Afterward, the participants were asked to comment on the questions' content, wording, and language. Furthermore, all the participants were asked if the questions addressed all of their urinary tract problems.

#### Factorial validity

Principal component analysis (PCA) was utilized to assess the homogeneity of the SF-Qualiveen questionnaire. In order to determine the appropriate sample size for factor analysis, the number of items in the questionnaire was taken into consideration. Recommendations suggest that a minimum of 3–20 samples per item is required, resulting in a range of 24–160 samples for factor analysis [20–22]. Additionally, another recommendation states that a minimum sample size of *n* = 100 is necessary for factor analysis [23]. Therefore, our sample of *n* = 108 satisfied both recommendations. To ensure sampling adequacy, the Kaiser–Meyer–Olkin (KMO) measure was employed, with values greater than 0.5 considered acceptable and values over 0.8 considered meritorious [24, 25]. The Bartlett's test of sphericity (BTS) was also conducted to confirm the suitability of conducting an exploratory factor analysis (EFA) on the included items. The factor solution was determined based on eigenvalues greater than 1 and the scree plot, which aided in determining the necessary number of factors to establish factorial validity. Factor loadings were evaluated, with values of ≥ 0.32 considered acceptable communalities, ≥ 0.5 indicating moderate communalities, and ≥ 0.8 representing high communalities [26, 27]. A minimum of 60% variance was considered acceptable for explaining the total item variance [28].

#### Setting and recruitment of the patients

Between September 2022 and March 2023, 143 individuals from four neurorehabilitation centers in the Syrian Provinces of Damascus and Latakia were enrolled in the study. These facilities specialize in treating people with diseases or nervous system injuries and providing physiotherapy, occupational therapy, and speech and swallowing therapy. Each center has a unit for addressing defecation and urination problems caused by neurological injuries. The physical therapy sessions are carried out by qualified therapists under the supervision of physiatrists. All subjects were over 18, diagnosed with spinal cord injury, and could read and speak Arabic proficiently. Patients who had undergone urologic surgery within the past 12 months, recently experienced a change in their overall state of health, and experienced a change in their neurogenic bladder (NB) or active urinary infection symptoms within the previous 30 days were excluded.

#### Instrument

Patients independently completed the NBSS-SF and SF-Qualiveen questionnaires, while the treating physician clarified the precise aim and content of the questionnaires' items. During this phase of the study, baseline demographic information was also collected. All the patients were required to come back after 2 weeks to re-complete the SF-Qualiveen questionnaire.

The SF-Qualiveen [14] is a short version of the Qualiveen-30 [6] questionnaire that assesses the quality of life related to lower urinary tract dysfunctions. The SF-Qualiveen comprises eight items organized into four domains of two items each: bother with limitations, fears, feelings, and frequency of limitations. The domains of this questionnaire include a variety of emotional, physical, and social issues related to urinary and bladder dysfunction. This instrument uses a 5-point Likert scale, with 0 denoting “no impact” and 4 denoting “high impact.” The overall score is the mean of the eight separate scores, with a higher value reflecting a lower QoL.

The NBSS-SF questionnaire [28] is a self-reported instrument developed to assess the symptoms of NB and evaluate the consequences of lower urinary tract dysfunction. This scale comprises 10 items organized into four domains: consequences (two questions), incontinence (three questions), and storage and voiding (three questions). Two additional questions, the first one is about the bladder management technique, and the second question is related to how the present bladder management technique impacts the quality of life. The overall score of the NBSS-SF ranges from 0 to 28, with higher values indicating greater severity of symptoms, in addition to the availability of the NBSS-SF in Arabic [29]. All the patients were invited to complete the questionnaire within 2 weeks to assess test–retest reliability. The interval between both tests was large enough to lower the likelihood that the patients would forget the questions and short enough to avoid any alters in the patients' bladder management techniques or symptoms.

### Statistical analysis

The data are shown as percentages when indicating mean and categorical data, while the standard deviation was employed for continuous data. Cronbach's alpha was computed to evaluate the SF-Qualiveen's internal consistency, with a value of 0.7 or higher being acceptable (from 0.00 to 0.49 = unacceptable; from 0.50 to 0.59 = poor; from 0.60 to 0.69 = questionable; from 0.70 to 0.79 = acceptable; from 0.80 to 0.89 = good; and from 0.90 to 1.00 = excellent) [30]. The intraclass correlation coefficient (ICC) was employed to measure test–retest repeatability. The construct validity of the questionnaire was identified by evaluating the association between SF-Qualiveen and NBSS-SF using Spearman's correlation coefficient. The association’s strength was calculated as follows: (less than 0.10 = negligible; 0.11–0.39 = weak; 0.40–0.69 = moderate; 0.70–0.89 = strong; and 0.90–1.00 = very strong correlation) [31]. Factorial validity was examined through principal component analysis (PCA) to determine the homogeneity of the SF-Qualiveen questionnaire. The sampling adequacy was assessed using the Kaiser–Meyer–Olkin (KMO) measure, with a threshold of greater than 0.5 indicating acceptable sampling adequacy. Additionally, Bartlett's test of sphericity (BTS) was employed to confirm the suitability of conducting an exploratory factor analysis (EFA) on the included items. The significance level was fixed at 0.05, and all statistical analyses were computed using SPSS version 23.0.

## Results

One hundred and fifty-six patients with symptomatic urinary disorders were asked to participate in this validation study. Thirty-seven patients did not respond to the participation invitation. Eleven individuals were removed for several reasons: Two patients changed treatments during the test–retest interval, four refused to complete the second questionnaire, and five did not return the second questionnaire for unclear reasons. Finally, 108 SCI patients completed the second questionnaire (retest), an average of 15.7 ± 7.4 days after the initial set of SF‐Qualiveen, and were accepted in the ultimate analyses. Table 1 shows the participants’ demographic information and clinical characteristics, in addition to the time of neurological disease, level of injury, educational level, and type of bladder management.

**Table 1:** Demographic and clinical characteristics

Demographic and clinical characteristics	*n* (total = 108)
Gender	
Male	77 (71.29%)
Female	31 (28.71%)
Age (years)	
Mean (SD)	39.54 (± 11.34)
Median (min–max)	39 (18–69)
Education level	
Illiterate	–
Elementary school	4 (3.70%)
Middle school	35 (32.41%)
High school	41 (37.96%)
College or more	28 (25.93%)
Injury time (months)	
Mean (SD)	31.22 (± 11.6)
Median (min–max)	31 (11–73)
Level of injury	
Cervical	39 (36.11%)
Thoracic	36 (33.32%)
Lumbar/sacral	33 (30.57%)
ASIA grade	
A	20 (18.52%)
B	54 (50.0%)
C	34 (31.48%)
D	–
Patients mobility	
Fully ambulatory	8 (7.40%)
Limited walking without aid	53 (32.41%)
Walking with aid	52 (40.15%)
Wheelchair	13 (12.04%)
Urinary symptoms	
Duration of urinary symptoms (years)	8.2 ± 6.1
Manner of bladder emptying	
Normal voiding	32 (29.78)
Abdominal pressure	3 (2.77)
Total incontinence	2 (1.85)
Intermittent catheterization	61 (56.40)
Indwelling catheter	10 (9.20)

*SD* standard deviation and *min–max* minimum–maximum

## Reliability

### Internal consistency

The internal consistency for the overall SF‐Qualiveen showed a good internal consistency (Cronbach's alpha of > 0.8). The domains “Bother with limitations,” “Fear,” “Feeling,” and “Frequency of limitations” also demonstrated good internal consistency, with a Cronbach's alpha of > 0.7, as shown in Table 2.

**Table 2:** Internal consistency—Cronbach's alpha (*n* = 108 SCI patients)

	Test	Retest
SF-Qualiveen total	0.91	0.89
SF-Qualiveen subscales		
Bother with limitations	0.85	0.89
Fears	0.73	0.82
Feeling	0.80	0.82
Frequency of limitations	0.66	0.73

*Qualiveen-SF* Qualiveen–short form

### Reproducibility

The overall SF-Qualiveen reproducibility was good, with ICCs of 0.90. The ICC value for every SF-Qualiveen domain was higher than 0.7 (0.83 for the bother with limitations, 0.80 for fears, 0.84 for feeling, and 0.82 for frequency of limitations), indicating good reproducibility (Table 3).

**Table 3:** Reproducibility of SF-Qualiveen

	Test (mean ± SD)	Retest (mean ± SD)	Mean change (mean ± SD)	ICC
SF-Qualiveen total score	2.39 ± 0.63	2.42 ± 0.43	0.05 ± 0.44	0.90
Bother with limitations	2.56 ± 0.69	2.58 ± 0.70	0.01 ± 0.43	0.83
Fears	2.40 ± 0.58	2.42 ± 0.64	0.02 ± 0.42	0.80
Feeling	2.84 ± 0.75	2.82 ± 0.91	− 0.02 ± 0.37	0.84
Frequency of limitations	2.56 ± 0.65	2.56 ± 0.43	0.00 ± 0.23	0.82

*SD* standard deviation, *ICC* intraclass correlation coefficient, and *LOA* limits of agreement

### Validity

#### Content validity

Face-to-face interviews with 30 patients with SCI were conducted to assess content validity. Most patients agreed that all items were necessary to examine the wide range of bladder problems that patients encounter. The questionnaires were typically accessible, simple to comprehend, and quick to complete for the participating patients, and no changes were required.

#### Construct validity

The construct validity was assessed using Spearman's rank test, which compared the SF-Qualiveen questionnaire to the SF-NBSS questionnaire. A significant strong association was observed between the QoL item of the NBSS-SF and the SF-Qualiveen overall score (*r* = 0.82, *p* 0.003) and bother with limitations domain of the SF-Qualiveen (*r* = 0.76, *p* 0.004). There was a substantial moderate positive association between the overall scores on the NBSS‐SF and the domains of the SF-Qualiveen, involving bother with limitations (*r* = 0.53, *p* = 0.02), fears (*r* = 0.44, *p* = 0.03), feelings (*r* = 0.49, *p* = 0.04), and frequency of limitations (*r* = 0.46, *p* = 0.02). The majority of the SF-Qualiveen domain demonstrated a moderate association with the quality of life and the storage and voiding domains. The results of SF‐Qualiveen showed weak correlation scores for the consequences domains of NBSS-SF (Table 4).

**Table 4:** Correlation between SF-Qualiveen and NBSS-SF scores

		NBSS-SF, total score	NBSS-SF, incontinence domain	NBSS-SF, storage, and voiding domain	NBSS-SF, consequences domain	NBSS-SF, quality of life item
Qualiveen-SF, total score	*r*	0.651**	0.421	0.624*	0.347	0.820**
	*P*	0.005	0.756	0.045	0.137	0.003
Qualiveen-SF, bother with limitations domain	*r*	0.531*	0.682	0.711*	0.362	0.762*
	*P*	0.022	0.241	0.02	0.092	0.004
Qualiveen-SF, fears domain	*r*	0.447*	0.471	0.422*	0.415	0.572*
	*P*	0.031	0.242	0.04	0.311	0.04
Qualiveen-SF, feeling domain	*r*	0.493*	0.437	0.485	0.432	0.711*
	*P*	0.047	0.112	0,632	0.092	0.023
Qualiveen-SF, frequency of limitations domain	*r*	0.461*	0.361	0.327	0.371	0.362
	*P*	0.029	0.147	0.241	0.083	0.133

*Qualiveen-SF* Qualiveen–short form and *r* Spearman's rho correlation coefficient

∗Significant correlation at *p* < 0:05 (bilateral)

∗∗Significant correlation at *p* < 0:01 (bilateral)

**Fig. 1:**
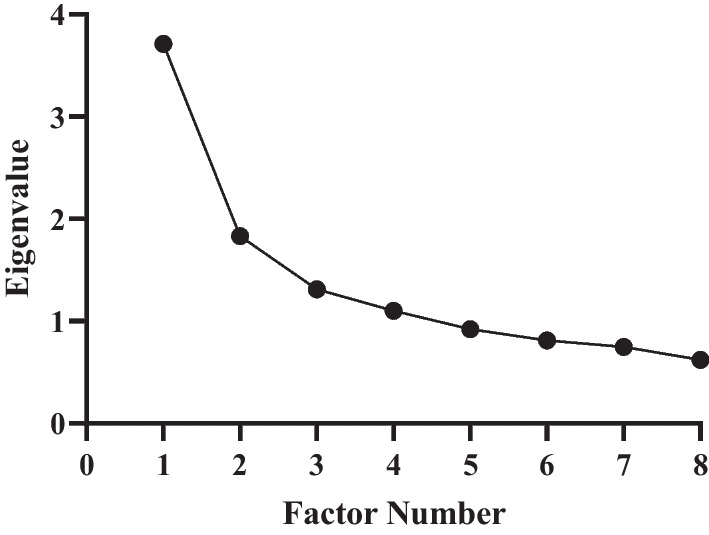
Scree plot confirming the factor number with eigenvalue (> 1)

The sample adequacy, with a sample size of 108, was confirmed to be meritorious based on a KMO measure of 0.83 [32,]. The Bartlett's test of sphericity (BTS) confirmed that the 8-item model was suitable for conducting principal component analysis (PCA), with a significant Chi-square value of 363.243 and p-value less than 0.001. Based on the criteria of eigenvalue greater than 1 and the scree plot (Fig. 1), the four-factor model was found to be a better fit for explaining the data compared to the 8-item model. The four-factor model accounted for a cumulative variance of 72.556%, which was considered acceptable (Table 5) [28].

**Table 5:** Four-factor model of principal component analysis for eight items

Items	Eigenvalues		
	Total	% of variance	Cumulative percentage of variance explained
1	3.712	33.251	33.251
2	1.834	17.461	50.712
3	1.317	12.427	63.184
4	1.112	9.372	72.556
5	0.926	8.193	80.749
6	0.831	6.831	87.580
7	0.751	6.537	94.117
8	0.623	5.883	100.000

Table 6 presents the factor loadings for each item of the SF-Qualiveen questionnaire. The minimum factor loading observed was 0.324, while the highest was 0.821. These factor loadings fall within the permissible range, confirming the factorial validity [33]. Table 7 displays the item communalities, ranging from 0.552 for the sixth item to 0.867 for the eighth item. These values indicate moderate to high communalities [20, 23]. Then, the homogeneity of the SF-Qualiveen questionnaire was determined using PCA, which provided evidence of factorial validity.

**Table 6:** Four-factor model with factor loading under eight items

Items	Component			
	1	2	3	4
1	0.821	0.728		
3	0.742	0.693	0.437	
5	0.727	0.623	0.371	
8	0.653			
2			0.351	
7	0.437			
6	0.321		0.629	0.731
4			0.324	0.529

**Table 7:** Factor loading of 11-item communalities

Items	Factor loading
1	0.631
2	0.682
3	0.622
4	0.673
5	0.571
6	0.552
7	0.613
8	0.814

## Discussion

The quality of life of patients suffering from chronic diseases such as spinal cord injury is a significant component of health care. The symptoms of lower urinary tract problems considerably affect these patients' quality of life [34]. As a result, employing questionnaires for patients with urinary symptoms is strongly suggested as an essential supplement to the diagnostic process [4, 5]. This study validated and culturally adapted the SF-Qualiveen after translating it into Arabic for use among SCI patients. The psychometric characteristics showed that the Arabic version of the SF-Qualiveen is a consistent, valid, and reliable instrument for use among SCI patients with lower urinary tract symptoms. We decided to validate and translate the SF- Qualiveen since it is practical, simpler to use in clinical practice and research, and less complicated for patients to complete. The length of the questionnaire was mentioned as a limitation in the previous studies, which evaluated the validity of the Qualiveen questionnaire [11, 12].

The SF‐Qualiveen was created to facilitate large-scale population surveys by reducing data-gathering time and cost [14]. The validations of the SF-Qualiveen in earlier research tend to be conducted with heterogeneous patient populations from various centers or patients with various neurological problems [10, 11]. Getting an effective and sufficient number of people with neurogenic disorders to conduct validation is difficult and often impossible. This study tried to validate the SF‐Qualiveen with the involvement of only SCI patients from four rehabilitation centers, providing a homogeneous sample of patients.

The Arabic SF-Qualiveen showed an internal consistency (Cronbach’s alpha = 0.91), similar to the previous validation studies among German SCI patients (Cronbach’s alpha = 0.89) [33]. The internal consistency of the feelings (0.80) and bother with limitations (0.85) domains was better than the frequency of limitations (0.66) and fears (0.73) domains. Our results are similar to the study conducted by Reuvers et al., where the internal consistency of the feelings (0.80) and bother with limitations (0.87) domains was better than the frequency of limitations (0.55) and fears (0.53) domains [33]. Regarding test–retest reliability after a median of 10 days, we found an ICC of 090, similar to those found in the SF-Qualiveen German (0.94) version validation study [33].

The association between SF-Qualiveen and NBSS-SF scores in patients with SCI has never been evaluated before. This association demonstrates the questionnaire's external validity and its relationship to clinical symptoms. The NBSS-SF is a frequently utilized evaluation tool in neuro-urology; nonetheless, it contains only one item about the QoL [32]. The SF-Qualiveen assesses life satisfaction in terms of lower urinary tract dysfunction. These two instruments complement one another when used in combination.

This study has some limitations, firstly inability to evaluate the responsiveness of the SF-Qualiveen, but other investigation studies have already established this characteristic [10]. Secondly, the lack of equality in the proportion of male and female participants led to limited generalizability by the ratio of male participants. Finally, all participants were recruited in four academic centers from two Syrian Provinces, but the participants in rural and distant locations may require a different approach. However, the questionnaire demonstrated good measurement characteristics in the original study and previous validation studies [14, 33].

## Conclusion

The outcomes of this study demonstrate that the SF-Qualiveen is a valid and reliable instrument for evaluating NDLUT-related quality of life in the Arabic population with spinal cord injury. Employing the SF-Qualiveen in their native language can help these patients and their physicians better comprehend bladder and urinary symptoms in regular practice. However, we need additional replication and validation of the results in greater, more varied populations. Future research should, therefore, concentrate on analyzing a large number of participants in the validation study and other psychometric features, like measurement invariance between groups with varied levels of education and health literacy.

## Data Availability

The data collected in this study are available from the corresponding author upon an adequate request.
